# Effect of Vitamin D Supplementation in Patients with Schizophrenia and Schizoaffective Disorder

**DOI:** 10.1192/j.eurpsy.2026.12164

**Published:** 2026-07-31

**Authors:** A. Jambrosic Sakoman, D. Bosnjak Kuharic, M. Herceg, S. Strkalj Ivezić

**Affiliations:** 1 Department for Psychotic Disorders- female; 2 University Psychiatric Hospital Vrapče, Zagreb, Croatia

## Abstract

**Introduction:**

Research indicates that the prevalence of vitamin D deficiency is higher among individuals with schizophrenia compared to the general population. Recent studies suggest that vitamin D deficiency is associated with an increased risk of various chronic diseases—including type 2 diabetes, hypertension, dyslipidemia, and cardiovascular disease—which are already highly prevalent in patients with schizophrenia. Vitamin D has also been inconclusively linked to cognitive impairment in this population, as well as to the severity of schizophrenia symptoms.

**Objectives:**

To examine the effect of vitamin D supplementation on cognitive function, positive and negative symptoms, and glucose and triglyceride levels in patients diagnosed with schizophrenia or schizoaffective disorder.

**Methods:**

We will include 40 female outpatients aged 18–65 years with diagnoses of schizophrenia or schizoaffective disorder. Assessments will include sociodemographic data (age, education level, body mass index, smoking, tea and coffee consumption, sun exposure, daily activity level, and diet); the *Brief Assessment of Cognition in Schizophrenia* (BACS) to evaluate cognitive functioning; the *Positive and Negative Syndrome Scale* (PANSS) to assess psychiatric symptoms; and laboratory measures of glucose and triglycerides. Data will be collected at baseline and after 12 weeks of supplementation. Patients will receive weekly oral vitamin D drops (25,000 IU).

**Results:**

We will analyze differences in outcome measures before and after supplementation.

**Conclusions:**

Vitamin D levels should be routinely monitored in patients diagnosed with psychotic disorders. Addressing vitamin D insufficiency through supplementation may be a cost-effective and clinically beneficial strategy.

**Disclosure of Interest:**

None Declared

